# Editorial

**Published:** 1869-05

**Authors:** 


### Editorial.

EXPLANATION.—On the 28th of April, when we left home to attend the meeting of the American Medical Association, the present number of the EXAMINER was so far printed, and the matter all furnished for its completion, that we supposed it would be sent to the subscribers promptly, as usual, during the first week in the month. Returning on the 15th of May, we were much surprised to find it still in the printer's hands. The delay had been occasioned solely by some misunderstanding in relation to the cuts with which the number is illustrated.

HOMŒOPATHY.—We have received a pamphlet of 100 pages, embracing four lectures on this subject, by A. B. Palmer, A.M., M.D., Professor of Pathology, Practice of Medicine, etc., in the Medical Department of the University of Michigan. These lectures embrace a very interesting and valuable exposition of the doctrines of homœopathy, as it was and is, and would be very useful for reference in the library of the physician, or even for general circulation among the people. Copies may be had at S. C. Griggs & Co., of this city. Price, 30c.

Illinois State Medical Society.—We are informed by the Committee of Arrangements that the members of the Illinois State Medical Society will commence in the Common Council Room, in the City Hall building, at 10 o'clock A.M., of Tuesday, May 18th, 1869. This is an excellent and convenient place in which to meet, and every arrangement will be made to promote the interests of the Society.

---

Correspondence.—Just after the last pages of our April number had gone to press, we received, in the form of advanced sheets, a copy of very interesting letters, written by Wm. O. Baldwin, M.D., President of the American Medical Association, and by G. C. Nott, M.D., formerly of South Carolina. The letters were written in excellent spirit, but related to matters bearing on the meeting of the Association this Spring, and, hence, could be of little use at this date.

---

Chicago Medical Society.—At the recent annual meeting of this Society, the following officers were elected, and committees appointed for the ensuing year:—*President*, R. G. Bogue; *Vice-President*, Ernst Smidt; *Secretary and Treasurer*, Hiram Wanzer. *Sanitary Committee*, Drs. Quales, Fenn, and Hutchinson. *Censors*, Drs. Fitch, McDonnell, and Holmes. *Committee on Ethics*, Drs. Davis, Trimble, and Margueret. *Committee on Microscopy*, Drs. Mitchell, H. M. Lyman, and Danforth. *Committee on Questions for Discussion*, Drs. Paoli, Hildreth, and Adolphus.

---

Adams County Medical Society.—The Adams County Medical Society, at its November meeting, adopted a revised Fee Bill, together with the following:—

In cases where consultations have been had, or where two or more physicians have been in attendance, the physician having the case in charge shall embody in his bill the fees of the counsel or assistants, and account therefor to the same, paying the full amount or such proportion of the whole bill as may be collected.

I. *Resolved*, That the foregoing table of fees be adopted as the Fee Bill of this Society.

II. *Resolved*, That the virtuous and industrious poor shall be attended by the members of this Society as cheerfully as the rich, and for such compensation as they are able to make.

III. *Resolved*, That every member of this Society shall keep a record of all patrons who are able, but who neglect or refuse to pay for medical services, and report the name and residence of the same at every meeting; and that all such persons be afterwards required, by all the members, to pay for services in advance.

IV. *Resolved*, That our fees are due as soon as the services are rendered, and that all members of the Society shall make prompt collections, and give the community to understand that we are entitled to the same consideration in this respect that is claimed by merchants, artisans, and laborers.

V. *Resolved*, That this Fee Bill, and these Resolutions, with the names of all the members of the Society appended thereto, be printed in a suitable form, and that every member be required to have a copy of the same conspicuously placed in his office, that our patrons may be fully apprised of its contents and conditions.

VI. *Resolved*, That members who disregard this Fee Bill and these Resolutions shall be liable to the same penalties that are attached to a violation of the Code of Ethics.

---

Money Receipts to April 26.—Drs. J. F. Kelsey, $3; D. A Sheffield, 3; S. J. Starr, 1; Daniel Gard, 3; John D. Wood, 3; D. S. Jenks, 3; Wm. Dougall, 3; A. G. Jones, 3; D. LaCount, 1.50; Harrison Rodbaugh, 3; J. B. Cloud, 3; R. Winton, 3; V. L. Hurlburt, 3; Theodore Hoffman, 3; Jessie H. Foster, 3; W. M. Chambers, 3; C. S. Hamilton, 3; J. H. Reynolds, 3; L. Brookhart, 3; Murphy and Wharton, 6.

---

The Discovery of a Minute Fossil Horse.—Professor Marsh, of Yale College, has discovered in the tertiary deposits of Nebraska, the minutest fossil horse yet obtained. It is only two feet high, although full grown. This makes the seventeenth species of fossil horse discovered on this continent.—*Boston Medical and Surgical Journal.*

Mortality for the Month of March, 1869:—

The sanitary superintendent submitted the following report of mortality for the month of March, 1869:—

The number of deaths during the month of March was 353, with 41 premature and still births.

COMPARISON.

| | | |
|---|---|---|
| Deaths in March, 1869,--353 | Deaths in March, 1868,--380 | Decrease,--- 27 |
| Deaths in Feb., 1869,------------382 | Decrease,--------------------- 20 | |
| Males, -----------207 | Females, -----------146 | Total, ---------353 |
| Single, -----------267 | Married ------------ 86 | Total, ---------353 |
| White, -----------349 | Colored, ----------- 4 | Total, ---------353 |

NATIVITIES.

| | | | | | |
|---|---|---|---|---|---|
| Bohemia, ---------- | 3 | France,----------- | 3 | Switzerland, ------- | 2 |
| Canada, ----------- | 8 | Germany,---------- | 33 | Sweden, ----------- | 8 |
| Native Chicago, ---- | 76 | Holland, ---------- | 2 | Scotland, ---------- | 3 |
| Foreign " ---- | 95 | Ireland, ---------- | 37 | Unknown,--------- | 2 |
| U. S., other parts,--- | 70 | Italy, ----------- | 1 | | |
| England, ---------- | 5 | Norway, ---------- | 5 | Total, ----------- | 353 |

MORTALITY BY WARDS FOR THE MONTH.

| Ward. | Mortality. | Pop. in 1868. | One death in | Ward. | Mortality. | Pop. in 1868. | One death in |
|---|---|---|---|---|---|---|---|
| 1--- | 6 | 9,094 | 2,273½ | 14--- | 24 | 14,839 | 742 |
| 2--- | 12 | 13,074 | 769 | 15--- | 28 | 21,078 | 958 1-10 |
| 3--- | 22 | 15,076 | 793½ | 16--- | 21 | 15,465 | 736 3-7 |
| 4--- | 18 | 17,796 | 936[illegible] | County hos. | 13 | | |
| 5--- | 19 | 16,033 | 943⅛ | Accidents, | 11 | | |
| 6--- | 18 | 13,083 | 769 1-10 | Mercy Hosp. | 5 | | |
| 7--- | 28 | 25,492 | 772½ | Suicides, | 1 | | |
| 8--- | 14 | 15,813 | 790 4-7 | St. Jo. Orph. | | | |
| 9--- | 27 | 19,297 | 689⅙ | Asylum | 1 | | |
| 10--- | 20 | 12,925 | 1,435 7-9 | Poisoning, | 9 | | |
| 11--- | 17 | 14,340 | 1,024 1-5 | Marine hosp. | 1 | Woman's home, | 1 |
| 12--- | 24 | 17,485 | 603 | Home for the | | Hosp. for Women & | |
| 13--- | 15 | 11,164 | 507½ | Friendless, | 2 | Children, | 1 |

Total, ---------------------------------- 374

REPORT OF SANITARY SUPERINTENDENT.

The sanitary superintendent also submitted his yearly report, of which the following is a brief synopsis:

The total number of deaths was 5,807. The sexes were, males, 3,191; females, 2,616. The condition was, married, 1,222; single, 4,585. Color, white, 5,742; colored, 65. In the way of nativities, Chicago suffers to the extent of 3,144. The greatest number of foreigners were from Germany, 671; the next greatest number, 510, being natives of Ireland. The mortality by wards shows the largest number to have been in the Seventh Ward, and the smallest in the First Ward. The total number of births was about 8,000. The number of females born during the year was much less than males.

At the last annual meeting of the Medical Society of the State of New York, Dr. March read a paper entitled "Spontaneous Lithotomy" giving an account of a case in which a large-sized vesical calculus was discharged through an ulceration in the perinæum.—*Medical Record.*

---

A Singular Disease.—Before the London Obstetrical Society, Mr. Heckford recently exhibited the generative organs of a child aged ten months, in which the vagina was enormously dilated, and occupied by villous growths of a medullary character. The rectum, bladder, and urethra were normal. The os uteri opened into the upper wall of the vaginal sac. The disease had lasted for about four months; and the child died shortly after its admission into the East London Children's Hospital.—*Medical Record.*

---

The Half-Yearly Compendium.—The number of this periodical for January has now appeared. It is full of the choicest selections on all branches of medical science, and should be in the hands of every physician. No synopsis of professional works, or articles, at all compare with it, either in the diversity of sources consulted, or the number of valuable facts accumulated.

The annoying delay in the publication of this number will be avoided in future. The next number may be confidently looked for by July 10th.—*Med. and Surg. Reporter.*

---

A Valuable Collection for Sale.—A large and superior collection of original illustrations, by the late Prof. Türck, of Vienna, comprising 777 water-color paintings of life size, and illustrating the diseases of the larynx and pharynx, as seen with the laryngoscope or from pathological specimens, is for sale by the family of the Professor. The paintings were executed by Drs. Elfinger and Heitzmann in the first style of the art. A history of each case is annexed. Will not some member of the profession so far render himself a benefactor of his brethren as to purchase the collection, that we may have it among us. F. H. B.—*Boston Med. and Surg. Journal.*

---

Catarrhus Vesicæ.—This disagreeable chronic complaint is often very obstinate; it may therefore, be stated that M. Mallez has found the following solution injected into the bladder very efficacious: Water ten ounces; tincture of iodine, forty-five drops; iodide of pottassium, fifteen grains. When the pain is very annoying, add fifteen grains of the extract of belladonna to the above. He has also employed carbolic acid, nitrate of silver, and hyposulphite of soda with advantage.—*Lancet, Jan. 2, 1869.—Medical News and Library.*

---

A Superior Liquid Glue.—A liquid glue, far superior to mucilage, may be made by dissolving glue in an equal quantity of strong hot vinegar, adding a fourth of alcohol and a little alum. This will keep any length of time when placed in closed bottles, and will glue together horn, wood, and mother of pearl. —*Scientific American.*

---

Diabetes Cured by Peroxide of Hydrogen.—Mr. J. J. Bayfield (*British Medical Journal*) reports a case of diabetes cured by peroxide of hydrogen. He commenced with half-drachm doses of the ethereal essence of the peroxide, and gradually increased it to three drachms a day.—*The Med. Record.*

---

A Surgical Prize.—The Surgical Society of Paris has just announced the subject of the Laborie prize (£48), to be awarded in January, 1870:—"Point out, by the aid of clinical facts, the actual value of supramalleolar amputation from the following points of view:—1. The mortality consequent upon the operation. 2. On the different ways of performing it. 3. The usefulness of stumps in the act of walking. 4. The artificial limbs best calculated for these stumps."—*Med. Record.*

---

Treatment of Diseased Gums.—A writer in the *London Lancet* recommends the following treatment of diseased gums: —The teeth should be washed night and morning with a moderately small and soft brush; after the morning ablution, pour on a second toothbrush, slightly damped, a little of the following lotion, and apply it to the affected parts:—Carbolic acid, one scruple; rectified spirits of wine, two drachms; distilled water, six ounces. By the use of this preparation, suppurative action is kept under, and the gums get firmer and less tender. —*The Medical Record.*

---

The Last Wonder of the Spectroscope.—The spectroscope, which, since its invention eight years since, by Bunsen and Kirchoff, has contributed so much to the progress of science, was used with signal success in observations of the recent total eclipse of the sun, by English and French parties, in different parts of Asia. By this means, the nature of the protuberances on the rim of the solar disc, observed in former eclipses, has been satisfactorily explained. They are found to be columns of incandescent gas, possibly containing hydrogen.—*The Medical Record.*

---

CALCIFICATION OF TOOTH PULP.—Recently Miss L., aged 21, of nervous, sanguine temperament and general good health, called for consultation in reference to her two superior central incisors. About four years before, they had received a blow which partially loosened them; they were quite sore and painful for a few weeks, and then recovered so far as to be used with a tolerable degree of comfort. The left tooth soon changed somewhat in color; and the presumption was that the pulps of both were devitalized. Two years and a half after the accident, the teeth began to change position, the cutting edges being thrown forward against the upper lip, disfiguring the mouth very much.

In consequence of this, together with constant soreness, which had existed for several months, it was decided to remove them, which being done nothing particular was observable, further than had been shown before extraction, the left tooth showing some change of color, but the right, none from that of a healthy tooth.

Through inadvertence, the crown of the latter, a day or two after extraction, was broken into three or four pieces, breaking off at the neck of the tooth; the pulp was found to be completely calcified, entirely filling the pulp chamber; it did not break; the fragments of the crown parted from it, leaving it standing perfect, tightly imbedded in the canal of the root so firmly that it can not be drawn out with the fingers. This is the only case of the kind we have ever seen, and is a very marked illustration of a process upon which very little attention has been bestowed, and about which not much is known.

We were recently presented by Dr. Cushing, of Chicago, with a section of tooth in which the calcification of the pulp was complete; but it was perfectly united to and continuous with the dentine all round the walls of the pulp chamber, which was obliterated thereby.

This is clearly a calcification of the pulp, and not a deposition merely of calcific matter upon the walls of the chamber, for the structure of the pulp is clearly seen in the tissue. We shall have sections of each mounted for microscopic examination, when we shall perhaps have something further to say in reference to them.

History of Vaccination.—The *Pall Mall Gazette* states that the Russian Government has offered a prize of 3000 roubles (£400) for the best history of vaccination, by way of celebrating the hundredth anniversary of the introduction of that practice into Russia by the Empress Catharine II. The prize is open to all European competitors, and the history may be written in any modern European language.—*Boston Med. and Surg. Journal.*

---

Webster's Unabridged—Illustrated.—In all the essential points of a good dictionary, in the amplitude and selectness of its vocabulary, in the fullness and perspicacity of its definitions, in its orthœpy and (*cum grano salis*) orthography, in its new and trustworthy etymologies, in its elaborate, but not too learned, treatises, of its introduction, in its carefully prepared and valuable appendices,—briefly, in its general accuracy, completeness, and practical utility,—the work is one which *none who read or write can henceforth afford to dispense with.*—*Atlantic Monthly.*

